# Review of atypical optic neuritis

**DOI:** 10.1007/s10072-024-07895-w

**Published:** 2024-12-18

**Authors:** Lepša Žorić, Emina Čolak

**Affiliations:** 1 Clinic for Eye Diseases, University Clinical Center of Serbia, Belgrade, 11000 Serbia; 2 Faculty of Medicine, UPKM, Kosovska Mitrovica, 38200 Serbia; 3 Institute of Medical Biochemistry, Scientific Research Department, University Clinical Center of Serbia, Belgrade, 11000 Serbia

**Keywords:** NMO spectrum disease, MOGAD, Systemic autoimmune diseases optic neuropathy, Neuroretinitis, Infectious and postinfectious optic neuritis, Postvaccination optic neuritis, Paraneoplastic neuritis

## Abstract

Optic neuritis (ON), an inflammatory optic neuropathy, is among the most common causes of visual loss. In its initial clinical appearance, ON may have unilateral or bilateral presentation, and anterior (papillitis) or retrobulbar localization. Traditionally, cases are divided into typical and atypical ON. In the Western hemisphere, most typical cases of optic nerve inflammation are associated with multiple sclerosis (MS). However, ON may also be associated with a series of disorders of known or initially undetected origin. Atypical ON has a somewhat different clinical picture from typical ON, and encompasses neuromyelitis optica spectrum disease (NMOSD), myelin oligodendrocyte glycoprotein antibody-associated disease (MOGAD), idiopathic recurrent neuroretinitis (NR), chronic relapsing inflammatory ON (CRION), ON within systemic autoimmune diseases, paraneoplastic and neuritis during or after infectious diseases or vaccination. The causes should be meticulously worked up, to address the therapeutic and prognostic challenges posed by these conditions. Here, we provide a brief overview of atypical ON, as encountered in our clinical practice, and additionally discuss the possible occurrence of optic neuropathies other than inflammatory and other ocular diseases within these disorders.

## Introduction

In patients with new-onset optic neuritis (ON), the first disease that may be suspected is multiple sclerosis (MS). However, since ON can be manifested in series of disorders, ON is traditionally divided into typical ON, a manifestation of MS, and atypical ON, which appears in other diseases.

ON is an inflammatory disease of the optic nerve, potentially involving its different compartments. Causes or triggering factors are numerous, and the mechanism of development is usually immunologically mediated, by triggering of an autoimmune reaction against certain components of the optic nerve. Less often, ON development is initiated by the presence of an infectious agent.

Disease diagnosis is based on patient history and basic ophthalmological examination findings. ON manifests as acute or subacute visual decline in one eye, or rarely in both eyes, color vision disturbances, some other visual disorders and, in some cases, pain with ocular movement. Further clarification and monitoring of the disease can be achieved through detailed ophthalmological, neurological, and radiological examinations, including visual field testing, pupil reaction testing (through investigation of relative afferent pupillary defect (RAPD)), color and contrast sensitivity testing, optical coherence tomography (OCT) of the ganglion cell layer or complex (GCL, GCC) and retinal nerve fiber layer(RNFL), sometimes OCT angiography (OCTA), magnetic resonance imaging (MRI) of the brain, orbit with optic nerves and spinal cord, and lumbar puncture and cerebrospinal fluid analysis. Although not necessary for ON diagnosis, electrophysiological testing (including visual evoked potentials (VEP), pattern reversal electroretinography (PERG), photopic negative response electroretinography (PhNRERG), and sometimes other electroretinography (ERG) methods) can aid in differential diagnosis and further monitoring of the disease [1–3]. Additional laboratory tests are used to rule out or confirm the suspicion of certain ON etiologies. For the same reason, consultations with other specialists may be required.

Therapy for ON involves immunosuppression. In new-onset ON, intravenous pulse corticosteroid therapy is usually used. Response to corticosteroids also aids in this classification. Further treatment, needed in relapsing, prolonged, cortico-dependent, and non-reactive cases, depends on the clinical course and underlying disease. Neurologists use various forms of immunomodulatory or disease-modifying agents [4]. In some defined ON, therapy is causal whenever possible.

## Typical optic neuritis

Typical ON is the most common presentation of the disease. ON within MS is more frequent in the Western hemisphere, whereas other demyelinating diseases (neuromyelitis optica spectrum disease (NMOSD) and myelin oligodendrocyte glycoprotein antibody-associated disease (MOGAD)) predominate in the Eastern hemisphere [5]. ON within MS largely defined attempts to classify and treat ON, which is not surprising, considering MS frequency and the substantial percentage of various forms of disability in individuals with some forms of MS.

Clinically, ON may manifests as a single episode (SION), or may become relapsing neuritis (RION). It can be first manifestation of MS or part of previously identified MS. Sometimes, it has a course of primary progressive ON. Mentioned forms of clinical course are not exclusively related to MS. However, each new case always needs more specific and differential investigations, because it could be a manifestation of other diseases.

Typical ON occurs most frequently in younger women. Affected individuals experience acute or subacute decline in visual acuity, pain in moving the eyes, scotoma in the visual field, and dyschromatopsia. The disease commonly occurs in one eye, in a form of retrobulbar neuritis. RAPD is present. MRI is necessary to examine the presence of demyelinating plaques, and to diagnose MS or assess the risk of its development or progression. OCT assessment, particularly of the GCC(L), may serve a similar function [6]. GCC(L) thinning, seen on OCT, may exist even without a prior clinically evident episode of ON. Further neurological evaluation includes lumbar puncture and cerebrospinal fluid analyses for oligoclonal bands.

Neuritis usually responds well to pulse corticosteroid intravenous therapy and commonly shows spontaneous recovery after several weeks [7]. In patients with MS, positive RAPD and extended p100 latencies on visual evoked potentials last several months or longer, after improvement of visual function.

## Atypical optic neuritis

Atypical ON has a more diverse clinical picture than typical ON. Optic nerve disease may include other optic neuropathies beyond inflammatory and may occur in several diseases, with differences in etiological, pathogenetic, anatomic, and clinical point of view.

The manifestations are often bilateral, in the form of papillitis, sometimes without pain during eye movement, and occur in all age and sex groups. The disease may have a progressive course and may show temporary or no improvement in response to corticosteroid therapy. The disease should be investigated through several modalities, and a diverse therapeutic approach is likely to be required (3, 4).

Atypical neuritis includes NMOSD, MOGAD autoimmune optic neuropathy, chronic relapsing inflammatory optic neuropathy (CRION), idiopathic recurrent neuroretinitis (NR), and optic neuropathy associated with systemic autoimmune diseases, infectious diseases, or vaccination [8]. Because atypical neuritis affects different parts of the optic nerve, and has various causes and varying clinical courses, its forms often overlap. Sometimes other non-inflammatory optic neuropathies (anterior ischemic optic neuropathy, hereditary optic neuropathy, compressive optic neuropathy and nutritional or toxic optic neuropathy) may have a similar course and presentation and it could be necessary to take them into consideration in the differential diagnostic work-up [9].

### Aquaporin-4 (AQP-4)-IgG-positive optic neuritis

ON within NMOSD is usually known from the best-described manifestation, Devic’s disease. In this autoimmune demyelinating disorder, inflammation of the optic nerve and the spinal cord (longitudinally transverse myelitis extending across three or more vertebral segments) may be accompanied by brainstem or brain symptoms (area postrema syndrome, diencephalic syndrome, or other brainstem disorders). Cases are more frequent in people not of European descent. Most patients meeting the current criteria for NMOSD experience repeated attacks separated by periods of remission, rather than a monophasic clinical course.

Damage to the afferent pathway at the starting point, at the level of ganglion cells (retina, examined by OCT or n95 wave of PERG and phnr wave on PhNRERG) are prominent but do not appear before ON, in contrast to ON in MS [10].

The interval between attacks may be weeks, months, or years. This condition can affect people of all ages, but the mean age of onset is in the early 40s, and cases predominate in females rather than males [11]. A blood test for antibodies against astrocyte water channel protein aquaporin-4 (AQP4-IgG) is highly specific and moderately sensitive for NMOSD. In illness, these antibodies are found in cerebrospinal fluid, and testing is frequently positive at the time of the first symptom, even before a confident clinical diagnosis is possible.

High intravenous corticosteroid doses are used as a first line treatment for acute attacks [12]. The prognosis for visual recovery is poorer in Devic’s disease than in typical forms of ON. Neurological monitoring and early intervention with plasmapheresis and other therapeutic immunosuppressive options are necessary to alleviate the deficits caused by this disease or even to prevent mortality [4, 13, 14].

### Myelin oligodendrocyte protein (MOG)-IgG-positive-antibody optic neuritis

A substantial portion of patients with another form of ON, frequently in a form of unilateral or bilateral papillitis, either isolated or associated with myelitis in adults, which phenotypically resembles Devic’s disease, but is AQP4-Ig negative—may instead be myelin oligodendrocyte glycoprotein (MOG) IgG positive [15]. In children, acute disseminated encephalomyelitis (ADEM), rather than myelitis, appears with ON.

MOG-ON is initiated by damage to the oligodendrocytes and is followed by demyelination; if other manifestations are present, it meets the criteria for MOGAD. MOGAD-ON occurs without previously recognized disease, but usually follows a viral infection or inoculation, such as bacterial or viral vaccination [16, 17].

The relatively recently discovered MOG-IgG is present in as many as half of patients with NMOSD who are AQP4-IgG negative. In the cerebrospinal fluid, this antibody is not normally present but is detected during the disease.

MOGAD usually has a less severe course than Devic’s disease. In the former, the optic nerve inflammation is more anteriorly located and affects a longer portion of the optic nerve [18]. Spinal cord lesions tend to be located in the thoracolumbar region, while ADEM is more frequent central manifestation in children [19]. Sometimes, the disease takes a course of CRION, including relapsing ON usually occurring 2 months after the end of corticosteroid therapy, and shows steroid dependency [13, 20]. Treatments for acute attacks entail high intravenous corticosteroid doses. Long term oral prednisolone therapy, for 3 months or more, has beneficial effects against relapsing disease. The absence of steroid-dependent attacks in early disease stages may predict a long-term non-relapsing disease course and relatively favorable outcomes [20]. Seroconversion to MOG antibodies negative finding is also a better prognostic sign [21]. In cases of MOGAD with progressive and damaging potential, other forms of immunosuppressive therapy are needed [4, 22].

### Idiopathic recurrent neuroretinitis (NR)

NR is characterized by acute visual loss with optic disc swelling, and macular star formation comprising hard exudates in the retina of one eye. The optic nerve is affected in the prelaminar part. The most common causes are infectious diseases due to *Bartonella henselae*, *Mycobacterium tuberculosis*, *Borrelia burgdorferi*, *Treponema pallidum*, and *Leptospira*. If the infectious cause is detected, antibiotics are included in therapy. The role of corticosteroids is therapy is not entirely clear.

NR may be associated with systemic autoimmune diseases. Some cases remain idiopathic, and respond well to intravenous corticosteroid therapy or spontaneous recovery without recurrence.

A subset of patients with NR has a different clinical profile characterized by larger visual field defects, moderate to large RAPD, and poor visual outcomes. The disease frequently affects both eyes, occurs in relatively young patients (mean age of 28 years), and has no sex bias. Poor therapeutic response and recurrence (two or more attacks) lead to progressive and permanent visual disability. The cause of this disorder has not been identified. Although laboratory testing has revealed no systemic disease in these patients, some researchers suspect an autoimmune disorder involving the optic disc vasculature. Corticosteroid therapy can address optic disc edema without visual improvement. Nonetheless, corticosteroids are usually included in treatments for initial attacks, and long-term low corticosteroid doses or other modalities of immunosuppressive therapy are needed for the prevention of new attacks [23, 24].

### Infectious and vaccine-associated optic neuritis

Atypical optic neuropathy may be associated with a wide variety of infectious agents. Causative factors include specific viral, bacterial, parasitic, or fungal diseases. Optic neuropathy may present as papillitis, retrobulbar ON, NR, perineuritis, and anterior ischemic optic neuropathy (AION). The disease can manifest as ON alone or show signs and symptoms of involvement of other parts of the nervous system. The mechanism of origin may be direct pathogen invasion, initiation of an autoimmune demyelination process, or vascular and coagulation disorders. Diagnosis is based on detailed history, serology, and additional analyses, which are frequently required (e.g., biopsy, lumbar puncture, polymerase chain reaction, protein immunoblotting, X-ray imaging, and MRI).

#### Viral neuritis

*Herpes simplex viruses* (*HSVs*) cause several ophthalmic diseases. The optic nerve is typically affected within acute retinal necrosis, during, before, or after retinitis, in a form of papillitis, and may cause profound vision damage [25]. Viral neuritis can occur during herpetic encephalitis. Rarely, ON may appear in one eye, and other herpetic eye diseases may be present in the other eye [26]. Diagnosis is based on HSV-1 and − 2 positive serology, and therapy relies on antiviral agents.

*Varicella zoster virus* (*VZV*) can cause bilateral papillitis in children, before, during, or after varicella onset. Visual outcomes are generally good. In zoster form, ON typically presents late (weeks to months after skin rash), and therapy involves a combination of acyclovir and corticosteroids [27]. During active zoster, possible ischemic optic neuropathy and neuritis within acute retinal necrosis or progressive outer retinal necrosis have been reported [28], and are more often found in immunocompromised patients.

*Cytomegalovirus* (*CMV*) retinitis and papillitis have been described primarily in patients with acquired immunodeficiency syndrome (AIDS) and those receiving immunosuppressive therapy for organ transplantation [29]. Several cases have been described in immunocompetent patients, who have shown good recovery after antiviral therapy [30, 31].

*Epstein-Barr virus* (EBV) causes infectious mononucleosis, but is also associated with several malignances. EBV associated neuritis is generally bilateral and manifests as papillitis [32], and responds well to corticosteroid therapy.

*Human immunodeficiency virus* (*HIV*) optic neuropathy may be unilateral or bilateral, and may present as retrobulbar optic neuropathy, papillitis, ischemic optic neuropathy, or optic disc pallor [33].

Optic nerve inflammation in measles, rubella, influenza, and infections with West Nile virus, coronavirus, or other viruses, have been reported [17, 34].

#### Bacterial neuritis

*Mycobacterium tuberculosis* (*MTB*) may affect all ocular tissues [35–37]. Ocular tuberculosis (TB) is rare, even in immunocompromised individuals, and usually occurs without signs of active systemic disease. The disease develops after staying in endemic areas [36] or with the activation of latent TB in different of immunosuppressive states, including caused by immunosuppressive therapy.

The most common manifestations of ocular tuberculosis are granulomatous iridocyclitis, choroiditis and retinal vasculitis. The clinical spectrum of tuberculous optic neuropathy is broad, including papillitis, NR, optic nerve tubercles, compressive optic neuropathy, anterior ischemic optic neuropathy, optic atrophy, papillary edema in posterior scleritis and meningitis, and optic chiasmatic arachnoiditis. ON is usually unilateral and painless. Associated posterior uveitis or panuveitis are frequently observed. A definite diagnosis should be evidence of the presence of bacilli in the intraocular fluids and tissues, preferably by polymerase chain reaction (PCR). Due to the high risk of sampling ocular tissues, which makes them difficult or even impossible, the diagnosis is most often presumed, based on indicators of tuberculosis infection elsewhere in the body (quantiferon TB gold test, chest X-ray lung). Antituberculosis treatment is also challenging, especially considering the possibility of systemic use of corticosteroids, and known possible toxic effect of antituberculosis drugs, especially ethambutol, on the optic nerve.

Syphilis is a sexually transmitted disease caused by the *Treponema pallidum* spirochete. Its ophthalmic manifestations are numerous. Optic nerve involvement may present as papillitis, perineuritis, chiasmal syndrome, gumma of the optic disc, NR, and optic disc cupping. Optic neuropathy mostly occurs in secondary and tertiary syphilis, and any intraocular involvement is considered to indicate neurosyphilis, so CSF examination should be performed. The disease is known as the “great mimicker,” and these ambiguous manifestations are particularly clear in ophthalmology. Numerous tests for *Treponema pallidum* are available and treponemal specific serology tests are necessary [38]. As appearance of syphilis is often related to immunocompromised conditions, it is mandatory that all patients with newly diagnosed syphilis should be tested for human immunodeficiency virus (HIV). Penicillin is an effective drug when used according to the regimen for neurosyphilis, and the use of corticosteroids can ameliorate the disease.

*Borrelia burgdorferi* is the causative agent of Lyme disease [39]. Bacteria are transmitted to humans through tick bites, and the disease occurs in three stages. The disease may affect central and peripheral nervous system. Different ophthalmic manifestations are possible [38], and ON can have forms of papillitis, rarely retrobulbar neuritis and NR. Photophobia can be prominent symptom. Serology (ELISA) testing for IgM and IgG for Borrelia should be followed by immunoblot analysis. As optic neuropathy is not pathognomonic, and the data on the primary skin disease are not certain, this disease should be suspected in all cases of ON and serology ought to be done. The microorganism is sensitive to ceftriaxone or doxycycline treatment.

The most frequent causative agent of NR is *Bartonella henselae*. Cat scratch disease is transmitted by cat scratches or bites, and the resultant systemic illness is mild and similar to influenza, with tender lymphadenitis. This stage may go unobserved. The disease may evolve toward Parinaud oculoglandular syndrome or hematogenous spread, when neuroretinal and retinal disease may develop. NR manifests several weeks after contact with an animal and begins with unilateral optic neuropathy manifestation, which is clinically visible as papillitis. These symptoms are followed by retinitis, and a characteristic macular star composed of exudates develops on the posterior retinal pole 1 to 2 weeks later [40].

Mild anterior uveal reaction may occur. Diagnosis is based on clinical features and laboratory tests. Doxycycline is beneficial in more serious cases, whereas corticosteroid use is controversial. The disease typically has favorable outcomes, and may even resolve spontaneously, but visual acuity recovery occurs gradually over several weeks.

*Leptospira*, *Mycobacterium leprae*, *Tropheryma wippleii* (causing Wipple’s disease), *Brucella*, and other bacteria can rarely cause ON, although they should nonetheless be considered possible causes.

#### Parasitic and fungal infections

*Toxoplasma gondii* is a ubiquitous protozoan parasite with feline hosts. The prevalence of parasite in human population, as an intermediate host, is considerable, but uncommonly causes clinically manifest disease. The microorganism is obligatory intracellular parasite with neurotropic characteristic. It could be transferred from newly infected mother to the fetus, via placental blood flow, and if it is the case in later gestational phases and pregnancy went smoothly, characteristic neuroretinal scars in a form of rosette are observed later, during childhood, usually as a cause of poor vision. Cerebral calcifications could be observed by X-ray. Ocular toxoplasmosis usually involves retina and choroidea from an old adjacent retinal scar, but can develops as a new retinochoroiditis. Immunocompromised people are particularly prone to the development of ocular disease, including encephalitis, often as a result of a new infection. The optic nerve is involved in forms of isolated papillitis, NR, retrobulbar ON or associated with retinochoroiditis [41, 42].

Diagnosis is challenging if characteristic scars are absent and is based on serological testing and aqueous or vitreus samples testing for local antibodies production, eventually for protozoan DNA detection [41]. Management involves a combination of antiprotozoal medications and corticosteroids.

Toxocariasis is a zoonosis caused by *Toxocara canis* and *Toxocara cati* nematodes. The typical ocular involvement is retinal granuloma. Several cases of optic neuropathy in the form of papillitis, retrobulbar ON, or NR have been reported [43]. Optic disc granuloma is a possible manifestation, usually with associated vitritis. As in ocular toxoplasmosis, diagnosis is established through serology, and rarely through additional aqueous humor or vitreous analysis, and calculation of the Goldmann-Witmer coefficient. Treatment is based on corticosteroids, whereas the role of anthelmintic medications remains controversial.

Mucormycosis is an opportunistic infection caused by *Mucorales* fungi, which are widespread in nature. Although infection is uncommon, it may occur in immunocompromised hosts, including people with uncontrolled diabetes mellitus. The disease has lethal potential. Rhino-orbital and rhino-orbito-cerebral mucormycosis include optic nerve infarction and necrosis. Invasion of blood vessel walls by the organisms leads to occlusion or thrombosis of the optic nerve sheath, blood vessels, or ophthalmic artery. Direct optic nerve infection by mucormycosis may also occur. The disease has become particularly relevant in the COVID-19 pandemic, in people with diabetes and those receiving corticosteroid therapy [44]. Treatment involves aggressive surgical debridement of all involved tissues, sometimes including exenteration of the involved orbits, and prolonged administration of amphotericin B.

*Cryptococcus neoformans* and other fungi can damage the optic nerve. Neuro-ophthalmic manifestations have also been described within other parasitic infections [34].

### Postvaccination neuritis

Postvaccination optic nerve inflammation is rare but well known. Beyond ON, postvaccination inflammation may be due to ADEM, transverse myelitis, or NMOSD in individuals with AQP4 and MOG antibodies, and MS, either as a new disease or relapsing previously diagnosed MS. They occur primarily after influenza and human papillomavirus vaccination, but also after immunization against other viruses and some bacteria [45, 46]. Suggested mechanisms include molecular mimicry between myelin basic protein and viral proteins, epitope spreading, bystander activation, and superantigen activation.

Since 2019, COVID-19 has been a dominant viral disease reaching pandemic proportions. Beyond the neuro-ophthalmological manifestations of the disease, with the development and widespread use of vaccines in disease prevention, postvaccination complications have been reported [47]. Postvaccination neuritis usually has a mild course, appears within 1 day to 1 month after vaccination, and may recover spontaneously or with pulse corticosteroid therapy, which accelerates visual recovery.

### Autoimmune diseases

Optic neuropathy can occur in numerous systemic autoimmune diseases (AID) and is often unrelated to lesions in other parts of the nervous system. Its etiology and pathogenesis are not fully understood, and most explanations are based on pathogenic mechanisms of the underlying disease. Sometimes defined as autoimmune optic neuropathy [48], this condition has diverse emerging forms that are not specific to certain diseases. It should be borne in mind that some types of biological therapy in AID may cause ocular side effects; particularly important are anti TNF α agents, which sometimes initiate ON and demyelination processes [49].

#### Granulomatosis with polyangiitis or Wegener’s granulomatosis

Granulomatosis with polyangiitis (GPA) or Wegener’s granulomatosis is a multisystem autoimmune disease affecting small and medium sized blood vessels. The upper respiratory tract, lungs, and kidneys are most frequently affected. This condition can occur at any age but usually appears in people in their 40s and 50s, and shows no sex bias. Inflammatory indicators, such as the erythrocyte sedimentation rate and C-reactive protein, might be elevated. Diagnosis involves imaging and laboratory tests, but a tissue biopsy of the upper respiratory tract or kidney is essential for definitive diagnosis. Positive serology findings for cytoplasmic anti-neutrophil antibodies (C-ANCA or anti-proteinase3 PR3-ANCA) are highly diagnostic for Wegener’s granulomatosis, whereas perinuclear ANCA (P-ANCA, against myeloperoxidase) may exist.

Ocular disease may be the presenting or dominant manifestation in patients with granulomatosis with polyangiitis, and can affect almost all parts of the eyeball, adnexa, and orbit. Thus, treating ophthalmologists should have a high index of suspicion, particularly in cases in which other features of the disease, such as pulmonary or renal disease, are absent [50].

The optic nerve may be affected in several ways. Optic neuritis, as papillitis or retrobulbar neuritis and perineuritis, ischemic optic neuropathy, and optic nerve compression by granuloma, have been described, with or without signs of orbital involvement [50–53]. Optic nerve ischemia is a result of focal vasculitis of small arteries, arterioles and small veins, vascular thrombosis and hemorrhage, or granulomatous inflammation, or may be a sequela of chronic inflammation.

Therapy is based on aggressive immunosuppression with high doses of corticosteroids and other immunosuppressants. Prolonged immunosuppression is required.

#### Systemic lupus erythematous

Systemic lupus erythematosus (SLE) is a chronic autoimmune connective tissue multisystem disease. Characteristic manifestations exist on the skin of the face and mucous membranes. The kidneys, and frequently joints and the central nervous system, are affected. The median age of onset is between the late teens and early 40s, and a significantly higher incidence is observed in women than men [54]. Major histocompatibility complex genes, such as HLA-A1, B8, and DR3, as well as alleles that cause deficiency in the complement components C1q, C2, and C4, have been associated with lupus. Numerous autoantibodies are detected in SLE [55]. Specific serology markers that may aid in the diagnosis of suspected lupus optic neuropathy are antinuclear antibodies (ANAs), anti-double-stranded DNA-ANA, anti-Sm, anti-nucleosome, anti-NMDA receptor, and anti-phospholipid antibodies [56].

Optic nerve disease, a rare manifestation of SLE, consists of ON, ischemic optic neuropathy, or NMOSD. However, ON can be the first manifestation of SLE [57, 58].

Optic nerve disease may manifest as a thrombotic, vaso-occlusive event with focal axonal necrosis, usually in one eye, and may be associated with antiphospholipid antibodies, or may appear as a general immunological inflammation, such as vasculitis and NMOSD. Poor presenting and final visual acuity and visual field defects are characteristic. The timing of inflammation treatment is important and should usually occur within first 10 days, because ON responds well to high dose corticosteroid treatment. Relapses are possible and require prolonged oral tapering or additional immunosuppressive agents [59].

#### Sarcoidosis

Sarcoidosis a multisystem granulomatous disease. Diagnosis is based on chest X-ray, in which hilar lymphadenopathy is present in most cases, and on angiotensin converting enzyme levels in the blood. Findings of hypercalcemia and calciuria, and changes in skin in the form of lupus pernio may aid diagnosis. Definitive diagnosis is established through biopsy and a finding of noncaseating granuloma.

Sarcoid granuloma may develop on different parts of the eye and adnexa. Neurosarcoidosis develops in approximately 5–15% of cases, and half of cases show neuro-ophthalmic manifestations. Visual loss results from visual pathway or optic nerve involvement. Sarcoidosis of the optic nerve can produce a variety of optic disc appearances. The optic nerve involvement may manifest as a swollen optic disc with hemorrhages, or as retrobulbar neuritis, NR, or optic nerve head granuloma [60, 61]. The optic neuropathy in sarcoidosis is typically painless or associated with mild pain, and the visual loss is subacute [62]. Involvement may be unilateral or bilateral. At presentation, similarly to other autoimmune diseases, optic atrophy may be observed, usually in one eye.

Granulomatous iridocyclitis or uveitis is relatively frequent manifestation, as for the other granulomatous disease, or even MS.

Systemic corticosteroid therapy remains the most rapid and effective initial treatment. Corticosteroids must be tapered over months to years. Steroid-sparing agents should be considered.

#### Sjögren’s syndrome

Sjögren’s syndrome (SS) is a chronic autoimmune disease characterized by periductal lymphocytic infiltration of the secretory exocrine glands, particularly the salivary and lacrimal glands [63]. The disease can exist alone, as primary SS, or on a background of other autoimmune diseases, as secondary SS.

Optic neuritis is not frequent but may be the first symptom of the disease. The clinical course is acute or chronic. Relapses are frequent in one or both eyes. NMOSD can be another manifestation of SS. In suspected cases of neuritis, the signs and symptoms of keratoconjunctivitis sicca and screening of blood anti-Ro/SSA and anti-La/SSB should be performed; in the context of NMOSD, anti-AQP4 antibody testing may be helpful. Biopsy of small salivary glands usually confirms the diagnosis [64]. Patients show minimal to moderate response to systemic corticosteroids or steroid dependence. Plasmapheresis in the acute phase and immunosuppressive agents for maintenance therapy are usually required.

#### Rheumatoid arthritis

Rheumatoid arthritis (RA), an autoimmune disorder affecting the joints, can occur with other autoimmune diseases [65]. Optic neuritis is rare in people with RA and usually appears late in the disease course. Approximately one quarter of people with RA have vasculitis with involvement in veins and arteries of all sizes. Occlusion of one of the posterior ciliary arteries or its branches produces ischemic optic neuropathy. Milder optic neuropathy can be inflammatory, and arise from vascular inflammation mechanisms and demyelination, and patients respond well to pulse corticosteroid therapy. More severe and irreversible cases of optic neuropathy experience axonal necrosis [66].

Optic perineuritis within RA, such as in other autoimmune diseases, has also been reported [52].

#### Behçet disease

Behçet disease (BD) is a rare disorder involving blood vessel inflammation in different parts of the body. The eyes are frequently involved. BD can have various optic nerve changes. ON can occur alone or with other CNS and ocular involvements, such as uveitis (neurouveitis), dural sinus thrombosis with papilledema, or obliterative retinal vasculopathy.

ON may be the first sign of the disease. Neuritis may be unilateral or bilateral, and potentially recurrent. The prognosis of BD-associated ON appears favorable, particularly if is the first presenting manifestation. Therefore, physicians should look for characteristic burdens and manifestations if disease is suspected (mucus membranes and skin efflorescence, joint pain, and other changes) and immunological analysis findings that may lead to a diagnosis (HLA-B51 major disease susceptibility gene, disordes of T cell immunity, interleukines).

Immunosuppressants should be administered along with corticosteroids [67].

#### Paraneoplastic optic neuropathy

Paraneoplastic optic neuropathy (PON) is a rare autoimmune disorder characterized by painless, progressive, typically bilateral, vision loss. Frequent association of spectrum of neurological disorders with ON raises suspicion to paraneoplastic disease [68]. PON panel of biological biomarkers should be done, and CRMP5 (earlier CV2) is particularly useful as one of the firstly detected paraneoplastic antibodies. Protein is located on oligodendrocytes, retina, and some parts of peripheral and central nervous system. An underlying cancer is usually diagnosed after ON appearing and the most frequent malignancy is small cell lung cancer, then ovarian, lymphatic or breast cancer [69, 70]. The most common age of patients is middle-aged or older. Therapy involves oncological treatment, along with corticosteroids and other immunomodulatory agents.

Frequently are present clinical signs of both retinopathy and optic neuropathy as autoimmune-related retinopathy and optic neuropathy (ARRON). It is found primarily in women, and usually appears in individuals in their 50s. Detection of antibodies against recoverin protein, which is found in the photoreceptors, as well as against α-enolase, Muller cells, glutamic acid decarboxylase (GAD), CRMP5-IgG and other PON biomarkers in serum have been reported in cases of ON or presumed ARRON [69, 70].

ARRON is diagnosed through exclusion, and autoimmune diseases, and cancer- and melanoma-associated retinopathy (CAR and MAR) should be ruled out. In cases with dominant optic nerve findings, MRI should be performed. Sometimes, other autoimmune diseases exist in patients. The prognosis may include moderate improvement in visual function or gradual worsening over months and years.

ERG abnormalities may be seen on full field and multifocal ERG, thus confirming retinopathy. However, the ERG features are not specific for ARRON, except some ERG characteristics in MAR. OCT shows thinning of the macular retina and photoreceptor cells [71] and visual field constriction.

### Chronic relapsing inflammatory neuropathy

Chronic relapsing inflammatory neuropathy (CRION) and its biological markers correspond primarily to MOG-IgG positive antibodies ON. The association between these entities has not been fully evaluated. Both are described as isolated disease, in postinfectious and postvaccine courses and within systemic autoimmune diseases [16, 72]. CRION is the most frequent manifestation of the relapsing ON associated with MOG-IgG [72, 73]. Patients with bilateral papillitis relapsing ON who show steroid dependency in the absence of the AQP4-Ab must be tested for MOG-IgG. Among patients with MOG-IgG-associated ON, the absence of steroid dependency in early disease stages may predict favorable outcomes. A relapsing course requires prolonged oral corticosteroid therapy or administration of other immunomodulatory drugs [4]. Beyond optic nerve inflammation, most cases show no MRI lesions or show only nonspecific white matter abnormalities. Relapsing neuropathy results in functional failure and leads to atrophy and morphological changes that are best shown on OCT.

## Conclusion

Every ON presents a challenge. Atypical ON may occur alone or together with multiple diseases, and may show markedly diverse clinical features, but not indispensable different from typical ON. The direction of diagnostic procedures may also depend on the stage of the disease at presentation as well as the clinical course. As most of atypical ON are not pathognomonic, and only few have specific serologic biomarkers, diagnosis is often achieved through exclusion of several possible triggers, through numerous tests. Some causes are not common and therefore are rarely considered. Moreover, most reports in the literature describe single cases or case series.

Herein, our goal was to briefly describe the most frequently encountered atypical ON manifestations and their characteristics. Therapy may be dependent on the clinical course, and may involve casual, short or long-term immunosuppression. The number of these inflammatory neuropathies encountered in daily practice is expected to increase and awareness of these conditions may be helpful to physicians and their patients.
